# Molecular and Genetic Basis of Inherited Nephrotic Syndrome

**DOI:** 10.4061/2011/792195

**Published:** 2011-09-06

**Authors:** Maddalena Gigante, Matteo Piemontese, Loreto Gesualdo, Achille Iolascon, Filippo Aucella

**Affiliations:** ^1^Division of Nephrology, Department of Biomedical Science, University of Foggia, 71121 Foggia, Italy; ^2^Nephrology and Dialysis Unit, Department of Medical Science, Research Institute, Casa Sollievo della Sofferenza Hospital, Viale Cappuccini n. 1, San Giovanni Rotondo, 71013 Foggia, Italy; ^3^Department of Biochemistry and Medical Biotechnology, University of Naples Federico II and CEINGE- Advanced Biotechnologies, 80127 Naples, Italy

## Abstract

Nephrotic syndrome is an heterogeneous disease characterized by increased permeability of the glomerular filtration barrier for macromolecules. Podocytes, the visceral epithelial cells of glomerulus, play critical role in ultrafiltration of plasma and are involved in a wide number of inherited and acquired glomerular diseases. The identification of mutations in nephrin and other podocyte genes as causes of genetic forms of nephrotic syndrome has revealed new important aspects of the pathogenesis of proteinuric kidney diseases and expanded our knowledge of the glomerular biology. Moreover, a novel concept of a highly dynamic slit diaphragm proteins is emerging. The most significant discoveries in our understanding of the structure and function of the glomerular filtration barrier are reviewed in this paper.

## 1. Introduction

The ultrafiltration of plasma during primary urine formation is one of the central function of the human kidney. Normal filtration function of the glomerulus depends on the structural and functional integrity of the filtration barrier, that is the primary target of several inherited and acquired glomerular disorders, characterized by nephrotic syndrome (>3.5 g protein/day) and rapid progression to end-stage renal disease (ESRD). 

The glomerular filtration barrier, responsible for the size and charge-selective properties of renal filter, is composed of three separate layers: the fenestrated endothelium, the glomerular basement membrane (GBM), and the podocyte foot processes layer. Recent studies have emphasized the role of podocytes as a key cell type involved in the mechanisms responsible for proteinuria and glomerular damage [1–3]. Podocytes are injured in many forms of human and experimental glomerular diseases, including congenital nephrotic syndromes, minimal change disease (MCD), focal segmental glomerulosclerosis (FSGS), membranous glomerulopathy, diabetes mellitus, and lupus nephritis [1, 3, 4]. In fact, the majority of glomerular diseases are characterized by alterations in the molecular composition of the slit diaphragm (SD) and a reorganization of foot process structure with fusion and effacement. The major causes leading to foot process effacement and proteinuria are (i) abnormalities in the GBM or podocytes-GBM interactions; (ii) impaired formation of the slit diaphragm area; (iii) alterations of the actin cytoskeleton and associated proteins [1, 5–7]. 

A better understanding of the molecular properties of GBM, podocytes and the slit diaphragm is critical to develop novel therapeutic strategies for patients with glomerular disease and to prevent end-stage renal insufficiency. 

**Figure 1:**
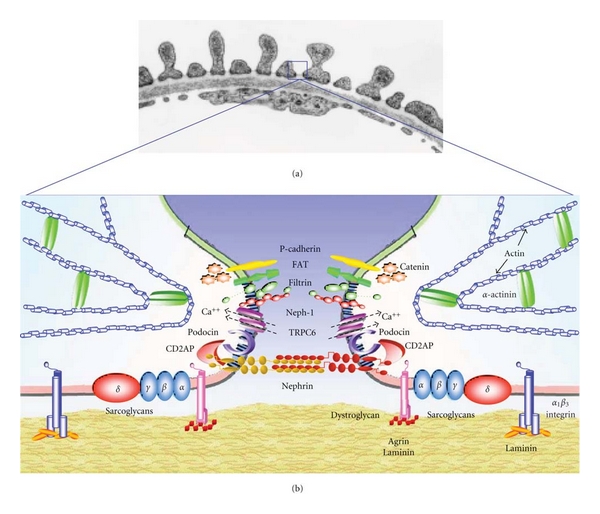
(a) Low-power view of glomerular filtration barrier in situ. The glomerular filter consists of three components: porous endothelium, glomerular basement membrane, and podocyte foot processes with the interposed slit membrane. Figure is transmission electron microscopy from a rat. Magnification: *B*, x~48,000. (b) Schematic drawing of the molecular equipment of the podocyte foot processes, similar to the area marked in Figure 1(a). See text for further explanations, (modified from [23]).

Mutations in different podocyte proteins can target the function of the podocyte through distinct pathologic mechanism by affecting the structure of the slit diaphragm, by directly or indirectly perturbing the intricate podocyte cytoskeleton, by breaking cell-matrix interactions, or by blocking important signaling pathways. All these mechanisms result in a common final disease pathway characterized by podocyte foot processes effacement, proteinuria, and ultimately disruption of the glomerular filter (Figure 1). 

The most significant discoveries in our understanding of the structure and function of the glomerular filtration barrier and related diseases are summarized in this paper.

## 2. Podocyte Structure and Development

Glomerular podocytes are highly differentiated cells with a complex cytoarchitecture. They have a voluminous cell body, long primary processes and regularly spaced, interdigitated foot processes that completely enwrap the glomerular capillaries (Figure 1). The interdigitated foot processes of neighboring podocytes cover the GBM and form a narrow filtration slit connected by an electron dense structure, called the slit diaphragm (SD), a zipper-like structure with a 40 nm diameter, according to the model proposed by Karnovsky and Ainsworth [8]. Podocytes are polarized epithelial cells with a luminal or apical and a basal cell membrane domain. A well-developed cytoskeleton accounts for the unique shape of the cells and the maintenance of the processes. The apical membrane of foot processes is equipped with a negatively charged surface coat, primarily made up of podocalyxin. This protein is critical for formation and preservation of cellular architecture: its absence causes immature glomeruli with flattened podocytes. The first marker of podocyte development in vertebrates is the restriction of *WT1* expression to a subset of cells within the renal vesicle [9, 10]. Several other transcription factors are expressed in the early podocytes, including podocyte-expressed 1 [11], forkhead box C2 (*Foxc2*) [12], kreisler (*Mafb*) [13], the forkhead domain transcription factor *Mf2* [14], and the Lim domain protein *Lmx1b* [15]. 

WT1 is probably the best studied of the transcription factors expressed in podocytes. *WT1* encodes a protein with four zinc fingers that can bind to both DNA and RNA [16, 17]. In the fetal kidney, WT1 is expressed in metanephric mesenchyme, renal vesicles, and developing podocytes. In adult life, the WT1 expression is restricted to podocytes. In maturing glomeruli, WT1 expression increases while the PAX2 expression is downregulated. The homeobox PAX2 gene encodes for a transcription factor expressed early during development and essential for conversion of metanephric blastema to renal vescicole. Downregulation of PAX2 appears as a prerequisite to allow podocyte differentiation which is governed by WT1. Both WT1 and PAX2 knockout mice lack kidneys, suggesting the critical role of these transcription factors in metanephric development. Mutations in PAX2 gene are associated with renal coloboma syndrome and isolated renal hypoplasia. The WT1 expression is altered in both congenital and acquired human diseases. In particular, the WT1 expression is lost in podocytes of collapsed glomeruli. Dominant mutations in WT1 are associated with the Denys-Drash and Frasier syndromes, characterized by glomerulopathy, mesangial sclerosis, male pseudohermaphroditism, and nephroblastoma. In these patients, the WT1 abnormal expression is associated with increased expression of PAX2 [18, 19]. 

POD1 (also known as epicardin and capsulin) encodes a basic helix-loop-helix transcription factor that is expressed early in mouse kidney development, and subsequently in the primitive podocytes of S-shaped bodies [20, 21]. Kreisler (MAFB) encodes a basic domain leucine zipper (bZip) transcription factor of the MAF subfamily and is expressed in mouse podocytes of capillary loop-stage glomeruli [22]. It also has an important role in hindbrain segmentation. *Pod1* and kreisler mutations in mice result in similar phenotypes: glomerular development is arrested at the single capillary loop stage [20, 22], and the podocytes remain as columnar-shaped cells that have lost their lateral cell-cell attachments but remain fully adhered to the GBM without any foot processes. Thus, *Pod1* and kreisler are required just prior to the time when podocytes would normally begin migrating around the capillary loops and assembling foot processes. *Pod1* is expressed in kreisler mutant podocytes, indicating that kreisler is likely to act either downstream or in a separate pathway from *Pod1* [22]. 

*Foxc2* was identified during a screen for genes with enriched expression in mouse glomeruli [12]. It belongs to the forkhead domain family of putative transcription factors and is expressed in podocytes. In *Foxc2* mutant mouse kidneys, mesangial cells cluster at the base of the glomerular stalk, podocyte foot processes, and endothelial fenestrations are absent, and dilated capillaries are observed, similar to the other phenotypes discussed above [12].

LMX1B, a Lim homeobox gene, is another important transcription factor, regulating the expression of multiple genes which are critical for podocyte differentiation and function. Homozygous Lmx-1b knockout mice have reduced numbers of podocyte foot processes, absence of typical slit diaphragms, and glomerular basement membrane abnormalities, but they express near-normal levels of nephrin, synaptopodin, ZO-1, and GBM laminins. Mutations of the human Lmx-1b gene are responsible for the nail-patella syndrome, an autosomal dominant disease with skeletal abnormalities, frequently associated with glomerulopathy [24].

## 3. Podocyte-GBM Interactions

Proteinuria, the most common clinical manifestation of glomerular diseases, is invariably associated with podocyte foot process effacement, flattening, and retraction. To maintain the complex foot process architecture, the adhesion of the podocytes to the GBM is controlled by the expression of several adhesion proteins. The foot processes are fixed to the GBM via *α*_3_ *β*_1_-integrin and dystroglycan (DG) complex. The *α*_3_ *β*_1_-integrin binds to fibronectin, collagen IV, and laminin of GBM, and it is essential for maturation of podocytes, as shown by the loss of foot processes development in *α*_3_-deficient mice. The dystroglycan complex is connected to podocyte actin cytoskeleton (Figure 1) through urotrophin, and its expression is reduced in MCD but not in membranous nephritis and FSGS [25–27]. 

Podocyte detachment leaves the denuded GBM, and this may play an important role in the FSGS pathogenesis: podocytes have not proliferative capacity and cannot repopulate denuded areas, and a scar is formed by parietal ephitelial cells [7]. 

The glomerular basement membrane (GBM), responsible for the charge-selective property of glomerular filtration barrier, is organized as a highly cross-linked network of specific extracellular matrix proteins, such as type IV collagen, fibronectin, laminin, nidogen, and heparansulfate proteoglycans (HSPGs). The flexibility and dynamism of the GBM requires a constant turnover. In the adult glomerulus, the podocytes continue to add and assemble GBM components and secrete matrix modifying enzymes [43]. 

Genetic modifications of structural GBM proteins, such as type IV collagen, cause Alport syndrome (AS), a hereditary nephropathy associated with deafness [30]. Thickening, basket-wave splitting, and rarefaction of the GMB have been reported in other hereditary nephritis with Döhle-like inclusions in polymorphonuclear cells and/or thrombocytopenia with giant platelets. This condition is known as Alport-like syndrome or Fechtner syndrome (FTNS), when Döhle-like bodies are associated with macrothrombocytopenia (MTCP) and Epstein syndrome (EPTS) when no leukocyte inclusions are present. Recently, it has been shown that mutations in MYH9, the gene encoding for nonmuscle myosin heavy chain IIA (NMMHC-IIA), are responsible for Fechtner syndrome, Epstein syndrome, and other two MTCPs (Sebastian syndrome and May-Hegglin anomaly) without renal, ocular, or hearing defects (Table 1). 

In the glomerulus, MYH9 mRNA and its protein are highly expressed by podocytes and colocalized with actin and *α*-actinin, suggesting that NMMHC-IIA could be an important component of the podocyte actin-myosin contractile apparatus and play a central role in maintaining capillary wall integrity [44–48].

## 4. The Slit Diaphragm Complex

Our knowledge of the molecular and structural composition of podocyte slit diaphragms have been improved in the past few years. The discovery of several novel slit diaphragm proteins, including nephrin, podocin, Zonula Occludens 1 (ZO-1), CD2-associated protein (CD2AP), P-cadherin, catenins, FAT1, Neph1-3, densin, and TRPC6 [33–38, 40, 41, 49, 50], has helped to characterize the region of the SD as a critical locus of podocyte function (Figure 1). Moreover, mutations in the genes encoding for slit diaphragm proteins have been linked to a variety of inherited and sporadic glomerular diseases (Table 1).

The first protein located at the SD domain is nephrin, codified by *NPHS1* (19q13.1), the gene responsible of congenital nephrotic syndrome of the Finnish type (CNF), a rare autosomal recessive disease with a highest incidence in Finland [51–53]. Nephrin is a large transmembrane protein of 1241 amino acids, belonging to the immunoglobulin (Ig) superfamily, with eight extracellular Ig-like motifs, a fibronectin type III-like, a transmembrane, and an intracellular domain [54, 55]. 

Nephrin is a critical structural component of the slit diaphragm complex: both CNF patients with severe NPHS1 mutations and knockout mice fail in foot process and slit diaphragm development and exhibit severe proteinuria already in *utero* [56]. 

Recently, Donoviel et al. identified *NEPH1*, a novel nephrin-like protein, that localizes to the slit diaphragm and causes congenital nephrotic syndrome in knockout mice. NEPH1 belongs to a family of three closely related proteins (NEPH1, NEPH2, NEPH3) with a common domain architecture [39]. 

Ihalmo et al., independently, identified NEPH3 gene, which they called NLG1 (nephrin-like gene 1). NLG1 or NEPH3 was localized to chromosome 19q13.1, immediately adjacent to the NPHS1 gene, and encodes a type I transmembrane protein, termed *filtrin*, which contains an extracellular region with five tandem immunoglobulin-like domains, a transmembrane region, and a cytoplasmic domain with a proline-rich region. 

Another important protein of the slit diaphragm complex is *podocin*, an integral protein, homologous to the band-7 stomatin family [34]. Podocin is codified by NPHS2 (1q25–1q31), the gene responsible for autosomal recessive steroid-resistant nephrotic syndrome [34, 57, 58]. Due to its structural similarity to stomatin, podocin is predicted to have a hairpin-like membrane topology, with both NH_2_- and COOH-terminal intracellular domains. Podocin expression is restricted to podocytes. Podocin localizes to the podocyte foot process membrane and accumulates in an oligomeric form in lipid rafts of the slit diaphragm. Podocin associates with CD2AP and nephrin via its COOH-terminal domain (Figure 1). These findings suggest that podocin may have a crucial role in the assembly of the SD complex, and, similar to the role of stomatin in erythrocytes, it may act as a scaffolding protein [23, 59, 60]. 

A complex of nephrin, podocin, and CD2AP seems to be indispensable to maintain the structural integrity of the SD. CD2AP, initially described as a protein involved in T-cell activation, contains a coiled coil domain and three Src homology 3 (SH3) domains, which serve as attachment sites for other proteins. In CD2AP, knockout mice immune function was compromised and they died of massive proteinuria shortly after birth, suggesting an important role in glomerular function. Knockout mice presented flattened podocytes, mesangial cell hyperplasia, and extracellular matrix deposition. Mice with CD2AP haploinsufficiency developed glomerular changes at 9 months of age and had increased susceptibility to glomerular injury by nephrotoxic antibodies or immune complexes. Interestingly, some glomerular lesions of these mice exhibited a phenotype similar to human FSGS [61]. Kim et al. screened a population of 30 African Americans with idiopathic FSGS and 15 African Americans with HIV-associated FSGS for changes in CD2AP. Six distinct DNA variants, absent in control subjects, were detected in 10 of 45 patients. One nucleotide variant, altering the exon 7 splice acceptor site, was predicted to alter the expression of CD2AP. These findings and others implicate CD2AP as a determinant of human susceptibility to glomerular disease [35, 62–64]. 

Nephrin, podocin, and CD2AP are pivotal for slit diaphragm structural organisation, suggesting that these proteins could participate in a common cell-signaling pathway. 

Huber et al. have demonstrated that both nephrin and CD2AP interact *in vivo* with the p85 regulatory subunit of phosphoinositide 3-OH kinase (PI3K). PI3K is the first protein demonstrated to interact with the cytoplasmic surface of SD protein complex *in vivo*. Nephrin and CD2AP recruit PI3K to the plasma membrane and, together with podocin, stimulate PI3K-dependent activation of the serine-threonine kinase, AKT. They demonstrate that nephrin-induced AKT mediates phosphorylation of several target proteins in podocytes. Although the importance of this signalling is not fully understood, it is interesting that one target of nephrin-CD2AP-induced phosphorylation is Bad, a proapoptotic protein of the Bcl-2 family; its phosphorylation and inactivation protect podocytes against apoptosis, suggesting that the nephrin-CD2AP-mediated AKT activity can regulate complex biological programs. These findings reveal a novel role for the slit diaphragm proteins and demonstrate that nephrin, CD2AP, and podocin proteins, in addition to their structural functions, initiate PI3K/AKT-dependent signal transduction in glomerular [65, 66]. 

In addition to these characterized podocyte proteins, the slit diaphragm area contains several other components, including P-cadherin, Zonula Occludens 1 (ZO-1), FAT, and densin. P-cadherin is associated with signalling proteins *α*-, *β*-, *γ*-catenins. It colocalizes with the zona occludens associated protein (ZO-1), a member of the membrane associated guanylate kinase (MAGUK) family. FAT is a large cadherin homologue, localized to the slit diaphragm domain. FAT knockout mice exhibit perinatal lethality. The relation between P-cadherin, FAT, and nephrin is not known [67]. Densin is a new podocyte protein that belongs to the LAP protein family, characterized by leucin-rich repeats and PDZ domains. LAP proteins are involved in maintenance of cell shape and the apical-basal polarity, thus densin may be necessary for maintenance of podocyte polarity [50]. Finally, two recent studies by Winn et al. [37] and Reiser et al. [38] have identified six families with autosomal dominant hereditary FSGS caused by six different mutations in the gene encoding TRPC6, a nonselective cation channel. These mutations lead to a kidney disease with late onset and a variable rate of progression to FSGS. TRPC6 is a member of the transient receptor potential (TRP) family of nonselective cation channels [68, 69]. TRP channels have been implicated in different biological functions such as cell growth, ion homeostasis, mechanosensation, and phospholipase C-dependent calcium influx. Calcium, as a second messenger, affects many of these cellular functions. Will et al. have reported, in all affected members of a New Zeland family with autosomal dominant FSGS, a TRPC6 missense mutation, p.P112Q, within the first ankyrin repeats of the protein. TRPC6^P112Q^ mutation increased peak calcium concentrations after stimulation with diacylglycerol and also potentiated angiotensin-II-mediated calcium signaling in HEK293 cells. The authors have speculated that the enhanced calcium signaling conferred by the TRPC6^P112Q^ mutation might disrupt glomerular cells function or cause apoptosis and amplify injurious signals triggered by ligands such as angiotensin II, that promote kidney injury and proteinuria [37]. These findings are also consistent with the results reported by Reiser et al.: two out of five mutant proteins exhibited larger current amplitudes that wild-type TRPC6 channels. Using immunofluorescence and immunoelectron microscopy, Raiser et al. found that TRPC6 protein is enriched in podocytes and localized in podocyte foot processes near the slit diaphragms; moreover, TRPC6 interacts with nephrin and podocin but not with CD2AP [38, 69]. Moreover, we have recently demonstrated that *TRPC6* mutations can also be detected in children with early onset and sporadic SRNS and described for the first time a *de novo TRPC6 *mutation in a severe form of pediatric collapsing glomerulosclerosis [70]. 

Thus, TRPC6 might belong, together with nephrin and podocin, to a signaling platform located at the slit diaphragm domain, suggesting a possible involvement of TRPC6 channels in regulating the dynamics of foot processes and slit diaphragm (Figure 1).

## 5. The Slit Diaphragm Genes and Congenital Nephrotic Syndromes: Genotype/Phenotype Correlation

The discovery of mutations in the genes coding for slit diaphragm proteins in patients with inherited nephrotic syndrome (NS) has been a breakthrough in both molecular and clinical research of glomerular diseases [71–78]. Moreover, mutations in the slit diaphragm genes have been reported in sporadic cases [79, 80]. There is growing evidence that the presence of slit diaphragm gene defects has a great importance in clinical practice of nephrotic patients; in fact, the identification of mutations in nephrotic patients might allow the avoidance of unnecessary treatments, might permit the prediction of absence of recurrence after transplantation, and might allow for the provision of prenatal diagnosis to families at risk [81]. 

The best characterized inherited nephrotic syndromes are congenital nephrotic syndrome of the Finnish type (CNF) and Steroid Resistant Nephrotic Syndrome (SRNS), due to mutations in *NPHS1* and *NPHS2* genes, respectively. However, recently genes related to steroid-sensitive nephritic syndrome (SSNS) were also identified; finally, podocyte dysfunction is also seen as a component of several inherited multiorgan syndromes.

### 5.1. Congenital Nephrotic Syndrome of the Finnish Type (CNF)

CNF is an autosomal recessive disorder frequent in Finland (1 : 10,000), but it has also been described in various ethnic groups throughout the world [82]. The disease develops *in utero*, and a severe nephrotic syndrome, resistant to steroids or immunosuppressive drugs, is present from birth. Infants are premature with low birth weight and large placenta. Renal biopsy specimens show mild mesangial hypercellularity and extensive effacement of foot processes. Microcystic dilations of proximal tubules are common but not specific. Nutritional status and statural growth are poor, and children are highly susceptible to bacterial infections and thromboembolic complications. In patients with CNF who progress to ESRD between 3 and 8 years of age, the only long-term and life-saving treatment is renal transplantation [51, 52]. 

The CNF gene (NPHS1), encoding for nephrin, has been mapped to chromosome 19q13.1 by Kestilä et al. using the positional cloning approach [33]. In Finnish patients, two main NPHS1 gene defects, Fin-major (c.121delCT) and Fin-minor (p.R1109X), were found in over 94% of the CNF cases, suggesting the existence of two founder effects [53]. Recurrence of proteinuria after transplantation, as a result of the development of antinephrin antibodies, occurs in 20% of the patients with Fin-major/Fin-major genotype, which leads to the absence of nephrin in native kidney [83]. Outside Finland, CNF constitutes the commonest type of congenital nephrotic syndrome, but the exact incidence is unknown. Several non-Finnish cases emulate the classically severe clinical phenotype seen in Finland, and a variety of NPHS1 mutations distinct from Fin-major and Fin-minor have been detected [49, 74–76]. However, an unexplored area remains the milder disease phenotypes, with occasional remission of proteinuria [77]. It seems that NPHS1 mutations causing a total absence of nephrin expression and a complete flattening of foot processes are responsible for a severe, therapy-resistant form of nephrotic syndrome; while patients with NPHS1 mutations causing only partially defective nephrin may still have slit diaphragms and respond to therapy [73].

To date, about 173 different mutations have been reported both in Finnish and non-Finnish patients. These mutations include small deletions, insertions, nonsense, missense, splice site, and promoter variations, and they are distributed throughout the gene, emphasizing a functional requirement for both extracellular and intracellular domains. A surprisingly large number of NPHS1 mutations are missense resulting in single amino acid substitutions, all located at the extracellular domain, particularly within immunoglobulin domains “hot spot” [84]. 

Nephrin is a signaling molecule, which stimulates mitogen-activated protein kinases. Nephrin-induced signaling is greatly enhanced by podocin, which binds to the cytoplasmic domain of nephrin. Mutational analysis suggests that abnormal or inefficient signaling through the nephrin-podocin complex contributes to podocyte dysfunction and proteinuria [65].

### 5.2. Steroid Resistant Nephrotic Syndrome (SRNS)

Steroid-resistant NS is characterized by an autosomal recessive transmission, onset of proteinuria between 3 months and 5 years, resistance to steroid treatment, rapid progression to ESRD, absence of recurrence after renal transplantation, and absence of extrarenal disorders. Minimal changes on early biopsy specimens and FSGS at later stages are observed [58]. The causative gene, *NPHS2*, encoding for podocin, was mapped to 1q25–q31 by positional cloning approach [34].

NPHS2 mutations were first described in children with familial steroid-resistant idiopathic nephrotic syndrome. More than 116 pathogenic mutations have been found to segregate with the disease [85–87]. These mutations alter the expression of the gene or the structure of the protein. Two mutations, the R138Q and the R138X, were recurrent: the first one was observed in patients originating from Germany or The Netherlands, and the second one in families with Israeli-Arab descent. The R138Q podocin is retained in the endoplasmic reticulum and loses its ability to recruit nephrin in lipid rafts [88]. 

Podocin mutations have also been reported in patients with congenital or infantile nephrotic syndrome. Schultheiss et al. [89] found NPHS2 gene mutations in 11/27 (41%) patients with congenital nephrotic syndrome and NPHS1 gene mutations in 15/27 (55%) patients. Caridi et al. [90] reported an infantile steroid-resistant nephrotic syndrome associated with FSGS in three children with a homozygous haplotype in which two mutations are present in cis (P20L and R168H). Tsukaguchi et al. analyzed NPHS2 gene in 30 FSGS families with adolescent or adult onset. In six of these families, the affected subjects were compound heterozygous for R229Q amino acid substitution, which has an allele frequency of 3.6% in control population. Using *in vitro* translated podocin and purified nephrin, it was found that nephrin bound poorly to R229Q podocin; these data suggest that the R229Q mutation alone is, probably, insufficient to cause FSGS but it might enhance susceptibly to renal injury in association with a second NPHS2 mutation or variants in other genes, such as nephrin. However, the clinical relevance of the R229Q variant is unknown [91]. Pereira et al. [92] found that R229Q polymorphism was associated with a 2.77-fold increased risk of presenting microalbuminuria. It remains to be demonstrated whether this polymorphism is a risk factor for developing end-stage renal disease.

NPHS2 mutations have also been reported in 10 to 33% of sporadic steroid-resistant NS, which represents a frequent cause of ESRD in children [57, 79]. Ruf et al. [86] studied 152 patients with sporadic FSGS and found that 32 (21%) had homozygous or compound heterozygous mutations. Weber et al. [87] found a lower mutation rate of 6.4% in 172 patients with sporadic steroid-resistant nephrotic syndrome. 

Podocin mutations are restricted to steroid-resistant patients. In a recent study, no podocin mutations were found in 124 children with steroid-responsive nephrotic syndrome, confirming the results of Frishberg et al. and Caridi et al. [57, 78, 86]. The identification of podocin mutations in sporadic cases of steroid-resistant nephrotic syndrome is important for therapeutic decisions and genetic counseling. None patients with sporadic steroid-resistant NS and podocin mutations had complete remission following cyclosporine or cyclophosphamide treatment; only few patients presented a partial remission after cyclosporine therapy, but long-term benefit of this treatment is not documented, and cyclosporine may be nephrotoxic in patients with persistent proteinuria. Thus, steroid-resistant patients should be tested for podocin mutations before giving immunosuppressive therapy. Rapid screening of these patients for NPHS2 mutation is possible because of the small size of the gene. However, it should be remembered that not all familial cases of steroid-resistant nephrotic syndrome are linked to NPHS2 gene, indicating that other genes remain to be identified. This is important to understand therapy results and for a possible multicenter therapeutic trials.

Finally, Koziell et al. [77] detected NPHS2 mutations in two patients with typical CNF in whom NPHS1 mutations were not found and mutations in both NPHS1 and NPHS2 genes were found in four cases with congenital FSGS (di-genic inheritance). These data, confirmed successively by other studies [87, 89], indicate an epistatic gene interaction, resulting in a rare example of multiple allelic hits, and provide the first evidence for a functional interrelationship between nephrin and podocin. These findings demonstrate the genetic heterogeneity of congenital nephrotic syndrome and the absence of genotype/phenotype correlations. Congenital nephrotic syndrome may also be due to WT1 mutations and diffuse mesangial sclerosis. Currently, three genes are associated with congenital nephrotic syndrome: NPHS1, NPHS2, and WT1 [79, 93].

### 5.3. Steroid-Sensitive Nephrotic Syndrome (SSNS)

Whereas gene identification has furthered the understanding of pathomechanisms in steroid-resistant nephrotic syndrome (SRNS), not even a gene locus is known for SSNS. Total genome linkage analysis was performed in a consanguineous SSNS kindred, 11 patients, to identify a gene locus for SSNS. Homozygosity mapping identified a locus for SSNS on chromosome 2p12–p13.2 [94]. 

This locus is not responsible for the disease in all SSNS families, demonstrating that, like SRSN, this phenotype is also genetically heterogeneous. In fact, other authors reported an extended SSNS Bedouin family with a high rate of consanguinity. The clinical presentation and steroid response of its 11 affected individuals were similar to those of sporadic SSNS, but it was not linked to any of the presently known chromosomal loci nor predicted to be caused by mutation in any one of a list of genes associated with nephrotic syndrome [95].

### 5.4. Diffuse Mesangial Sclerosis

Children with diffuse mesangial sclerosis appear normal at birth, with a normal birth weight and without placental enlargement. The nephrotic syndrome may be present at birth or even suspected *in utero* by the finding of an elevated plasma alpha-fetoprotein level in the mother or the discovery of large hyperechogenic kidneys [96]. Abnormalities in the PLCE1 gene, which encodes phospholipase C epsilon, appear to cause isolated diffuse mesangial sclerosis. In one study of 12 children from 6 families with the disease, homozygous truncating gene mutations in PLCE1 were found in eight children [97]. Phospholipase C epsilon is a member of the phospholipase family of enzymes that catalyzes the hydrolysis of polyphosphoinositides resulting in generation of second messengers (e.g., inositol-1,4,5-triphosphate), which are involved in cell growth and differentiation. A pathogenetic role for PLCE1 in glomerular development was supported by findings of disruption of the glomerular filtration barrier and edema in a PLCE1 knockout zebrafish model. How a PLCE1 gene defect results in changes in the glomerular nephrotic syndrome is unknown. One possible explanation is that phospholipase C epsilon interacts with GTPase-activating protein, which is known to interact with the slit diaphragm protein, nephrin. Perturbations of this normal interaction would have a downstream effect including the subsequent interaction of GTPase-activating protein with nephrin.

## 6. Syndromic Disease

### 6.1. WT1 Mutations

The WT1 gene encodes a transcriptional factor of the zinc finger protein family that is involved in kidney and gonadal development. WT1 has been localized to chromosome 11q13; it consists of ten exons and generates four different isoforms resulting from alternative splicing [98]. After birth, WT1 protein expression is restricted to renal podocytes where it probably contributes to maintain an adult differentiation. Germline heterozygous WT1 mutations have been extensively reported in the literature as the cause of Denys-Drash (DDS) and Frasier (FS) syndromes that are characterized by nephrotic syndrome, genitalia anomalies, and pseudohermaphroditism. Renal findings in DDS are predominantly characterized by diffuse mesangial sclerosis of early onset and rapid evolution to end-stage renal failure, while FS usually presents slow progressive focal segmental sclerosis (FSGS). WT1 mutations associated with nephrotic syndrome are restricted to exons 8 and 9, that represent a sort of hot-spot that may be easily investigated. The three major studies [99–101] overall confirm an incidence of WT1 mutation in patients under 18 years around 6-7%. A most remarkable finding is that, in young females, this incidence is higher (10–12%) and probably becoming the most frequent inherited cause of nephrotic syndrome under 18 years in this sex cohort. Moreover, we have demonstrated that WT1 splice mutations are not rare in females under 18 years with SRNS, frequently in absence of phenotype change typical of Frasier syndrome. In adults and children with SDNS, screening analysis is of no clinical value. WT1 hot spot mutation analysis should be routinely done in children with SRNS; if the molecular screening anticipates any further therapeutic approach, it may modify the long term therapeutic strategy [101].

### 6.2. LMX1B Gene

LMX1B gene mutations are associated with autosomal dominant nail-patella syndrome, a condition displaying dysplastic nails, hypoplastic patellae, and glomerulopathy with proteinuria and hematuria [102]. Its phenotype is highly variable, and the main pathologic finding is an altered GBM. LMX1B gene is a transcription factor that plays an important role in glomerular development, regulating the transcription of multiple genes integral for proper glomerular basement membrane formation and/or glomerular podocyte differentiation and function [103]. LMX1B binds to the putative enhancer sequence of COL4A4, the gene for alpha-4 chain of collagen type IV [103].

### 6.3. LAMB2 Gene

LAMB2 gene mutations are associated with Pierson syndrome, an autosomal recessive syndrome characterized by congenital nephrotic syndrome with histologic lesions of diffuse mesangial sclerosis and ocular malformations (microcoria, abnormal lens with cataracts, and retinal abnormalities) [104]. LAMB2 gene encodes the laminin beta 2, a protein abundantly expressed in the glomerular basement membrane where it plays a role in anchoring and in the development of podocyte foot processes [105]. LAMB2 mutations have also been found in patients with congenital nephrotic syndrome and either no or less severe ocular abnormalities.

### 6.4. CD151 Deficiency

Recent work in humans has shown that the tetraspanin CD151 is essential for the function of the kidney, as mutations in *CD151* have been identified in three patients presenting with hereditary nephrotic syndrome leading to end-stage renal failure, pretibial bullous skin lesions, sensorineural deafness, and thalassemia. CD151 is part of the tetraspanin family of proteins that are ubiquitously expressed, membrane-embedded proteins that share a similar structure, and form dynamic complexes with each other and with integrins. Mice deficient in CD151 develop proteinuria, FSGS, and kidney failure [106].

### 6.5. SMARCALI Gene

Immunoosseous dysplasia is a rare autosomic recessive disorder that presents with spondyloepiphyseal dysplasia, renal dysfunction, and T-cell immunodeficiency [107]. This syndrome is caused by mutations in SMARCALI gene that encodes for a widely expressed protein involved in the chromatin remodelling. The renal involvement is characterized by proteinuria, FSGS, and renal failure.

### 6.6. Beta4 Integrin Mutation

The occurrence of congenital nephrotic-range proteinuria secondary to focal segmental glomerulosclerosis has been reported in an infant with epidermolysis bullosa and pyloric atresia (EB-PA) [108]. 

EB-PA is an autosomal recessively inherited disease manifesting in the neonatal period with blistering of the skin and mucous membranes, as well as congenital gastrointestinal abnormalities including esophageal, gastric, or duodenal atresia. Both lethal and nonlethal forms have been described. The condition is caused by mutations in the a6 and b4 integrin genes, which are expressed in the hemidesmosomes of stratified epithelia. Most cases of EB-PA are associated with mutations in the b4 gene, ITGB4, located on the long arm of chromosome 17 [109]. Mutations in the a6 gene, ITGA6, located on the long arm of chromosome 2, are less frequent [110].

In the case reported by Kambham et al., a novel mutation in exon 31 of the b4 integrin gene, ITGB4, was identified. Authors proposed that the b4 integrin gene mutation led to expression of a dysfunctional protein important in the maintenance of normal glomerular permselectivity and podocyte integrity. The development of nephrotic-range proteinuria, without full nephrotic syndrome, is consistent with the role of b4 as a minor podocyte integrin. The failure to detect proteinuria more frequently in EB may relate to early mortality and the unique effect of this novel b4 integrin mutation on podocyte function.

### 6.7. Renal Disease and Mitochondrial Genetics

Mitochondrial diseases can give rise to various syndromes or association, namely, neurologic and neuromuscular diseases, cardiac, renal, hepatic, hematological and endocrinic, or dermatological presentations. Renal dysfunction associated with mitochondriopathies is generally a rare event. The most frequent renal symptom is proximal tubular dysfunction with a more or less complete de Toni-Debre-Fanconi Syndrome [111]. A few patients have been reported with tubular acidosis, Bartter Syndrome, chronic tubulointerstitial nephritis, or nephrotic syndrome. Any mode of inheritance can be observed: sporadic, autosomal dominant or recessive, or maternal inheritance [111]. 

Three cases that presented with central and peripheral nervous system involvement and with NS secondary to FSGS in the first decade of life were associated to ubiquinone deficiency [112]. More recently steroid-resistant NS or neonatal renal failure has also been described in patients who bore inherited *COQ2 *mutations [113]. Taken together, these data allow identification of a new entity within the category of mitochondrial cytopathies, characterized by inherited *COQ2 *mutations, proliferation of dysmorphic mitochondria, and primary glomerular damage. This new entity may be defined as “*COQ2 *nephropathy,” because the kidney seems to be a primary target in some patients presenting with isolated renal symptoms [113]. *COQ2 *mutations cause a renal disease that is characterized by variable renal lesions and widespread proliferation of dysmorphic mitochondria in glomerular cells. The clinical picture can be heterogeneous, and neuromuscular symptoms may complicate the course of the disease. Early recognition of this new entity may be crucial, because clinical symptoms can improve after ubiquinone supplementation, and neurologic complications may be prevented [114]. 

FSGS lesions have already been associated with mutations in the mitochondrial genome (3243A3G in the tRNALeu(UUR) gene), which may cause isolated glomerular disease [114–116]. Podocyte damage secondary to inherited mitochondrial dysfunction may cause visceral cell depletion, accumulation of extracellular matrix, and ultimately sclerosis of the glomerular tuft. In other cases, the same mitochondrial disease seems to trigger epithelial cell proliferation (in particular podocyte proliferation), associated with GBM collapse. Whereas increased apoptosis of podocyte cells may explain the mechanisms underlying FSGS formation in mitochondrial cytopathies [117], it remains unclear why in some cases the pathway taken by injured podocytes leads to proliferative lesions [118]. 

Mitochondrial dysfunction and altered mitochondrial gene expression have also been documented in patients with NS secondary to nephrin mutations [119, 120] suggesting that, regardless of the initial insult, mitochondria play an important role in podocyte metabolism and may be actively involved in the pathophysiology of various forms of NS.

### 6.8. Limp2 Gene

Lysosomal integral membrane protein type 2 (LIMP-2), the product of the SCARB2 gene (MIM_ 602257), is a member of the CD36 superfamily of proteins [121]. The absence of this protein in mice causes urinary and neurological alterations, associated with impaired vesicular trafficking and distribution of apically expressed proteins [122]. A deficiency in LIMP-2 resulting from a nonsense mutation in the SCARB2 gene has been recently described in humans [123]. When in a homozygous state, the mutation was associated with progressive myoclonic epilepsy without intellectual impairment and a nephrotic syndrome with strong accumulation of C1q in capillary loops of the kidney, whereas healthy parents were heterozygous for the mutation. The main clinical features are nephrotic syndrome, normocytic normochromic anemia, and thrombocytopenia.

The histological analysis of the medullar zone in renal material revealed extensive tubular alterations with isometric vacuolization in distal and collecting tubules and the presence of granular material in cortical tubules without inflammatory infiltration deposits [122]. 

Berkovic et al. [124] described LIMP-2 mutations in three patients with action myoclonus-renal failure syndrome. Action myoclonus-renal failure syndrome (AMRF [MIM 254900]) is a lethal inherited form of progressive myoclonus epilepsy associated with renal failure. It typically presents at 15–25 years with proteinuria evolving into renal failure or with neurological symptoms (tremor, action myoclonus, seizures, and later ataxia). The renal pathology is of focal glomerulosclerosis, sometimes with features of glomerular collapse. The disorder was mapped to 4q13–21, and microarray-expression analysis identified SCARB2/Limp2, which encodes a lysosomal membrane protein, as the likely candidate. Mutations in SCARB2/Limp2 were found in all three families used for mapping and subsequently confirmed in two other unrelated AMRF families. The mutations were associated with lack of SCARB2 protein. The heterogeneous pathology in the kidney and brain suggests that SCARB2/Limp2 has pleiotropic effects that may be relevant to understanding the pathogenesis of other forms of glomerulosclerosis or collapse and myoclonic epilepsies. However, mutations in SCARB2 might account for unsolved cases of progressive myoclonus epilepsy (PME) without renal impairment, especially those resembling Unverricht-Lundborg disease (ULD) [125]. Finally, contrary to earlier proposals, LIMB 2 mutations share no features with Charcot-Marie-Tooth disease both at the clinical and neurophysiological levels [124, 125].

## 7. Podocyte Cytoskeleton and Familial FSGS

The major processes of podocytes have an abundant and dynamic cytoskeleton composed mainly of actin-rich microfilaments, containing several actin-associated proteins, such as myosin, synaptopodin, and *α*-actinin. Podocyte damage and proteinuria can result from cytoskeletal alterations too, rather than direct alterations in slit diaphragm proteins.

Kaplan et al. found linkage to chromosome 19q13, in three families with clear evidence of autosomal dominant inheritance of FSGS, with late onset. They analyzed the NPHS1 gene, located in this interval, and found no mutations associated with this disorder. By BLAST analysis, they considered ACTN4, encoding *α*-actninin-4, as candidate gene, and a mutational screening was performed in affected individuals of families. *α*-actninin-4, an actin-filament cross-linking protein (Figure 1), is highly expressed in glomerular podocytes and involved in nonmuscle cytoskeletal function. ACTN4 missense mutations were identified in affected members of each family. Mutant *α*-actinin-4 protein binds F-actin more strongly than wild-type *α*-actinin-4. These data suggest that mutations in ACTN4 gene might cause an increased affinity for actin filaments, and podocyte actin cytoskeleton may be altered in this group of patients. Interestingly, *α*-actinin-4 deficiency not only causes recessive glomerular disease, but also increases cellular mortality [36]. Thus, lesions which compromise cytoskeletal functions of podocyte appear to result in a slowly progressive loss of podocyte, the hallmark of FSGS. 

Consistent with this hypothesis are also the results reported by Winn et al. and Reiser et al. in two recent works, in which a familial late onset form of FSGS, linked to chromosome 11q and caused by mutations in the gene encoding TRPC6, was described [37, 38]. A failure in the receptor-mediated influx of Ca^++^ through mutated TRPC6 protein, a nonselective cation channel, might underlie the new 11q-linked FSGS. Cytoplasmatic calcium concentrations are tightly regulated to prevent cellular damage. The authors speculate that mutated TRPC6 channels might disrupt glomerular homeostasis and/or cause podocyte apoptosis. The onset of kidney disease linked to mutations in TRPC6 gene occurs at a relatively advanced age. There are two possible explanations of this finding: podocytes express several other TRPC channel subtypes, so late onset of disease might be caused by compensation for impaired TRPC6 function by other channels; in addition, TRPC6 mutations might cause only a minor damage in podocyte function and lead to irreversible alterations in the presence of a second glomerular insults [69, 126]. However, to better understand the exact function of TRPC channels in podocytes and their role in familial and acquired forms of FSGS, additional studies should be performed.

Recently, Brown et al. found heterozygous mutations in the formin *INF2* gene segregating with FSGS in 11 (12%) of 93 families with age at diagnosis and ESKD varying from 11 to 72 years and 13 to 67 years, respectively [127]. This finding has been successively confirmed by Boyer et al. in a cohort of 54 families with a glomerular proteinuric disorder of apparent AD inheritance and documented FSGS in at least one affected member. Missense *INF2 *mutations were found in nine families (28 patients), translating to a detection rate of 16.7% [128].

INF2 is a member of the formin family of actin-regulating proteins that accelerate actin polymerization [129]. To date, 15 mammalian formin genes have been identified, among which are the best studied diaphanous-related formins (DRF): mDia1, mDia2, and mDia3. In the C-terminal half, DRF proteins contain the forming homology domains FH1/FH2 and the diaphanous autoregulatory domain (DAD) region, whereas the diaphanous inhibitory domain (DID) is localized at the N-terminal half [4]. Interestingly, all of the 13 INF2 mutations associated to FSGS lie within the DID region of the protein [127, 128], and six of them are localized in the corresponding INF2 region of a mDia1 DID subdomain interacting with IQ motif-containing GTPase-activating protein (IQGAP1) [128]. IQGAP1 has been identified as a Dia1-binding protein that is necessary for its subcellular location [130]; it is also involved in actin cytoskeleton dynamics [131] and has been shown to interact with the podocyte proteins nephrin [132] and PLCE1 [133]. Although, the exact mechanism explaining how mutations in the INF2 gene may lead to a proteinuric phenotype remains unclear, and these first observations reinforce the idea of podocytes as dynamic structures that are extremely sensitive to alterations in the spatial or temporal regulation of the actin cytoskeleton. Thus, *INF2 *seems to be a major gene of AD FSGS. Screening for *INF2 *mutations, at least in exons 2 to 4, encoding the DID domain, should be strongly considered in patients with an AD familial history of FSGS, even before *ACTN4* and *TRPC6*.

## 8. The Other Side of the Moon: Immunopathogenesis of Idiopathic Nephritic Syndrome

Although recent genetic approaches have elucidated the disease pathogenesis through the discovery of several podocyte genes mutated in distinct forms of hereditary nephrosis, the molecular basis of minimal change nephritis syndrome and FSGS with relapse remains still unclear. In this setting, the immune system seems to play a critical role in the active phase of this disease through disturbances involving several cell subsets, mainly T cells. 

MCNS may be a systemic disorders of T-cell function and cell-mediated immunity [134]. Nowadays, it is quite clear that MCNS is the most common kidney disease associated with primary immunological disorders and that the sensitivity to steroid and immunosuppressive therapy is an important argument in favour of the immune origin of the MCNS [135]. Recent reports suggest a clonal expansion of CD8+ T cells expressing the memory T-cell marker, CD45RO [136]. and of CD4+ T cell expressing the CD25 antigen, the IL-2 receptor chain in long-lasting, active disease. Moreover, also the native immune system seems to be involved in MCNS, probably through the signalling pathway of the NF-*κ*B [137]. It has been shown that peripheral mononuclear cells, including T cells, exhibit high NF-*κ*B binding activity involving the p50/p65 complexes during relapses, which returned to basal levels during remissions [138]. There is a T-cell commitment towards a Th2 phenotype in MCNS that might explain why these patients often display a defect in delayed-type hypersensitivity response, suggesting an abnormal Th1-dependent cellular immunity [135]. 

Moreover, a second set of signals mediated by co-receptors is needed to promote T-cell proliferation, lymphokine secretion, and effector functions. In this setting, C-mip is as new discovered gene of unknown function, which is initially identified in T lymphocytes of patients with MCNS [139]. c-mip interferes at different levels of cell signaling and in particular is involved in the Th2 signaling pathway [140]. 

An hypothesis unifying T-cell disorders and podocyte dysfunction has recently been proposed by Zhang and coworkers [135]. The functional alterations allowing to nephritic proteinuria could result from the downregulation of transduction pathways playing a key role in slit diaphragm function such as the nephrin-mediated pathway. It is possible that the circulating factor is somehow linked to the NF-*κ*B pathway and that podocyte and immune cells might share the same molecular defect [135].

## 9. Conclusions

Recent molecular studies have allowed a better understanding of structure and function of glomerular filtration barrier. 

Nephrin seems to be the main member of the slit diaphragm, where it forms a zipper-like ultrafilter structure; podocin and CD2AP, instead, have probably the function to connect the cytoplasmic domain of nephrin to cytoskeleton and lipid rafts of the SD. Moreover, the latter findings on ACTN4, TRPC6, and INF2 proteins suggest an important role of cytoskeletal dynamics in the normal maintenance of podocyte function. 

Nephrotic syndrome is a genetically heterogeneous condition. In general, recessive mutations in *NPHS1*, *NPHS2*, and *PLCE1* are associated with more severe disease with earlier onset proteinuria and ESRD presenting in infancy and throughout childhood, although some milder cases have also been noted. By contrast, dominant mutations in *ACTN4*, *TRPC6*, and *INF2* are associated with milder disease with later onset proteinuria in the second decade and ESRD in the third and fourth decades of life.

This new important discoveries may provide useful guidance to the clinicians in deciding whether a course of immunosuppressive drug treatment is appropriate. However, additional molecular genetics and *in vivo* studies should be improved to apply this new knowledge for the development of comprehensive molecular diagnostic test and new mechanism-based therapeutic tools.

## Figures and Tables

**Table 1:** Glomerular inherited diseases.

Disease	Gene locus	Gene	Exons (n°)	mRNA (kb)	Protein	Animal model	References
Autosomal dominant renal coloboma syndrome (RCS)	10q24.3–q25.1	PAX-2	11	3.5 kb	Paired box gene 2 (PAX-2), transcription factor	PAX2−/− knockout mice: lack kidneys.	[28]

(i) Denys-Drash and Frasier syndromes (ii) Wilms tumor	11p13	WT-1	9	3 kb	Wilms' tumor 1 (WT1), transcription factor	WT1−/− knockout mice: lack kidneys.	Rose et al.; *Cell, *1990.

Nail-patella syndrome	9q33.3	LMX-1B	8	1.1 kb	M homeobox transcription factor 1, beta	LMX1B−/− knockout mice: reduced numbers of podocyte foot processes, absence of SD and GBM abnormalities.	[29]

Alport syndrome (AS)	Xq22.3	COL4A5	51	6.4 kb	Type IV collagen, alpha 5 chain	Canine X-linked hereditary nephritis: transcription of COL4A5 gene was reduced by a factor of 10 in the affected dog.	Barker et al.; *Science, 1*990 [30]

(i) May-Hegglin anomaly (ii) Sebastian syndrome (SBS) (iii) Fechtner syndrome (FTNS) (iv) Epstein syndrome (EPTS)	22q12.3–13.1	MYH9	40	7.2 kb	Nonmuscle myosin heavy chain IIA (NMMHC-IIA)	—	[31]

Minimal change disease (MCD)	3p21	DAG1	3	5.4 kb	Dystrophin-associated glycoprotein 1	DAG1−/− mice: developmental abnormalities.	[32]

CNS of Finnish type (CNF)	19q13.1	NPHS1	29	4.3 kb	Nephrin	NPHS1−/− knockout mice: nephrotic syndrome, perinatal lethality, and effacement of podocyte foot processes.	Kestilä et al.;* Molec Cell, *1998 [33].

Steroid-resistant NS (SRNS)	1q25–q31	NPHS2	8	1.8 kb	Podocin	—	Boute et al.;* Nature Genet, * 2000 [34].

Focal segmental glomerulosclerosis (FSGS)	6p12	CD2AP	18	4.6 kb	CD2-associated protein	CD2AP−/− knockout mice: compromised immune function and death of massive proteinuria shortly after birth.	Kim et al.;* Science, * 2003 [35].

Focal segmental glomerulosclerosis (FSGS)	19q13	ACTN4	21	2.9 kb	*α*-actninin-4	ACTN4−/− mice: progressive proteinuria, glomerular disease, death by several months of age.	Kaplan et al.;* Nature Genet*, 2000 [36].

Focal segmental glomerulosclerosis (FSGS)	11q21-q22	TRPC6	13	4.5 kb	Transient receptor potential channel 6	—	Winn et al.;* Science* 2005 [37]. Reiser et al.; *Nat Genet* 2005 [38].

Unknown	1q21–q25	KIRREL o NEPH1	14	9 kb	Nephrin-like 1 (NEPH1)	NEPH1−/− knockout mice: nephrotic syndrome, perinatal lethality, and effacement of podocyte foot processes.	Donoviel et al.;* Molec Cell Biol*, 2001 [39].

Unknown	19q13.1	NLG1/NEPH3	15	2.5/3.5 kb	Filtrin	—	Ihalmo et al.;* Biochem Biophys Res Commun, *2003 [40]. Sellin et al.;* FASEB J, *2003 [41].

Clear cell renal carcinoma	4q34-q35	FAT	24	14.7 kb	FAT tumor suppressor homolog 1 (Drosophila)	FAT−/− knockout mice: perinatal lethality.	[42]
